# Book Reviews

**Published:** 1832-11

**Authors:** 


### BIBLIOGRAPHICAL NOTICES.

*Medical Botany; or, Illustrations and Descriptions of the Medicinal Plants of the London, Edinburgh, and Dublin Pharmacopœias; including a Popular and Scientific Account of Poisonous Vegetables indigenous to Great Britain. With Figures drawn and coloured from Nature.* By JOHN STEPHENSON, M.D. F.L.S., Graduate of the University of Edinburgh; and JAMES MORSS CHURCHILL, F.L.S., Member of the Royal College of Surgeons, and Fellow of the Med.-Botanical Society of London. *New Edition.* Edited by GILBERT T. BURNETT, F.L.S. M.B. RI. R.C.S. &c. No. I.—8vo. John Churchill, London.

THE London Medical and Physical Journal expressed a favorable opinion of "STEPHENSON and CHURCHILL's Medical Botany," long before the present editor of both these works had the most distant idea of being in any way connected with either; and the justice of this judgment, which is on record in these pages, existing circumstances do not incline us to impugn. Indeed, the correctness of this opinion may fairly be assumed from the fact that a large impression, including reprints of many numbers, has been, in a comparatively short time, sold, and a new edition called for, which he is willing to believe will meet with the same reception as the last: for, in answering this demand, the spirited proprietor of the work has not contented himself with merely giving a reprint of the first, as a second edition, but, having applied to the present editor to superintend its progress through the press, has, at his suggestion, agreed to have several of the figures redrawn and re-engraved, many corrections made in those not requiring entirely new plates, and the whole more carefully coloured.

This work has been merely announced as a new edition of "Stephenson and Churchill's Medical Botany;" and it *bonâ fide* is such, as the advertisements declare it; and this any one who inspects it will perceive: for, besides the various corrections which the progress of science has rendered necessary, and which will be apparent to the most careless reader, the only number which has yet appeared contains eight additional pages of letter-press; the chapter on Croton being doubled in extent, and that on Belladonna having four pages added. Similar additions and corrections will, of course, be made in the other numbers, whenever they are required.

We should not have said thus much upon the subject, nor even have done more than announce the publication, had we not heard that a belief was prevalent that our new edition of "Medical Botany" would resemble a *soi-disant* NEW edition of a work with a somewhat similar title; and the letter-press of which, instead of being even a *reprint*, has been (at least three fourths of it) lying on the warehouse shelves for years. What we now say is wrung from us in self-defence, and our readers will take the observations in the spirit in which they are made. It matters not to us, individually, whether a single copy be disposed of or a thousand; but we should have noticed the work, and probably much more at length, had it been edited by other hands; and we know not why we should refuse its proprietor and publisher, who has not only endeavoured to improve it, but has lowered its price, that notice which he undoubtedly would have received had he intrusted it to a stranger's care.

---

*Essay on the Origin, Symptoms, and Treatment, of Cholera Morbus, and of other Epidemic Disorders, with a View to the Improvement of Sanitary Regulations.* By T. FORSTER, M.B., F.L.S., M.R.A.S. Corresponding Memb. of the Acad. Nat. Sciences at Philadelphia, &c.—8vo. pp. 62. Keating and Brown, London; Treuttell and Wurtz, Paris.

THE "absurd doctrine of contagion" has met with another redoubtable opponent in Dr. FORSTER; and really, after the "gyration of epitomes" amongst which he has whirled his unfortunate readers to arrive at the "lumen zodiacale" of September 1828, from which he dates the beginning of this epidemic period, we should not wonder if some have confessed themselves non-contagionists, in order to escape the necessity of finishing the pamphlet. We have, however, perused it, and we find that the arguments it contains may be almost reduced to a syllogistic form. About two thirds of the essay consist of attempts to prove what has never been denied, viz. that there are epidemics which are not contagious: this forms the major proposition; secondly, Dr. F. affirms that cholera is an epidemic disease; and, thirdly, he concludes that (therefore) it is not contagious. The fallacy of such a mode of argument need not be formally insisted upon; and even the illustrations which have been brought forward to support it are far from being the most happy that might have been selected. As to atmospheric agents having produced all the epidemics that have ever raged, from the plagues of Egypt to the present cholera, the position is untenable; notwithstanding the line which conveys the relevant information that

"A NEW VOLCANO *has broken out in the sea near Sicily,*"

has been printed at an awful distance from the other paragraphs, and rendered as emphatic as italics and capitals can make it.

There are some passages which the author seems to consider of peculiar importance; and, as we cannot make long extracts, we shall give these, although they will appear in rather a disjointed form; but such is the tenor of the whole.

"One thing with regard to epidemics ought to be particularly noticed, as pointing out a sort of progressive malignity in the infecting air; it will be found that epidemics of the milder sorts precede, follow in the train of, and also circumvade, the central pestilence: thus, after there have been various fevers in any given place, at length a more decided pestilence comes, and in its outskirts again the lesser epidemics prevail. Now, I ask, How is the circumstance to be explained, if we admit the origin of these disorders to be from contagion? Does not this gyration of epitomes round a central disorder of greater malignity, strongly bring to our mind the manner in which whirlwinds and storms (which are whirlwinds of greater extent,) usually take place; and force us to conclude, from analogy, that the morbific atmospheres in question may obey laws analogous to those of atmospherical phenomena, of which electricity is the agent. During the late central fever at Gibraltar, other places in its vicinity, on the continent, were afflicted with slighter epidemics: and on the present occasion, while the more severe symptoms of cholera morbus were successively afflicting Russia, Poland, and Prussia, its epitome appeared in France, Germany, and England, in the form of bilious diarrhœa. I could enumerate the same sort of thing in twenty or more instances. Now there is nothing in this that looks like contagion: it is, on the contrary, analogous to well-known facts in metereology, and reminds us of the gentle wetting which those get who are lightly touched by the skirts of a shower, while persons who happen to be under its centre are drenched to the skin with water. Again, when an electrified cloud passes over our heads, the effects produced on those situated under its central parts, differ from the influence exercised by its outskirts on the nervous system of persons who may be below them. I do not pretend, by this analogy, to infer that the direct agent in pestilence is some modification of electricity, though there are very striking facts which favor this opinion; but I contend that the cause is derived from some peculiar condition of the air." (P. 5.)

So much for the progress of epidemic cholera, when existing; now for the cause of its being in existence.

"I consider what I call the epidemic period as having begun as early as September 1828, when that extraordinary lumen zodiacale was seen, September 29th, to stretch across the heavens. I have traced a succession of atmospheric changes since that period: the spring of 1829 became remarkably unhealthy; the mortality in some countries was prodigious: and the cold of the summer, in parts of Europe, as extraordinary. I was at Spa in the end of May 1829, and I remember, while examining the substance thrown out by an earthquake there, I found the cold as great as in winter; and on the morning of the 8th of June there was ice on the puddles of water by the banks of the Meuse, near to the town of Namur. I learned also from couriers, that the cold was severe all along the Rhine, and even in Austria. At Louvain they told me everybody was more or less ill, and I heard the same at Aix la Chapelle: a warmer air began at Cambrai, and it was as warm as usual at Paris; but in Spain I find the cold was great. The winter of 1829-30, which followed, was one of unusual severity all over the world, even in the South of Spain, and in Africa snow lay on the ground, and in most parts of Europe covered it, from November 1829 to the end of February 1830. The cholera morbus then broke away from India, and began its deadly course towards Europe, but did not arrive in Russia till last spring. The plague, however, broke out at Jassy, and in Moldavia severe illness prevailed. During the present year, the cholera morbus has been making a certain progress, while milder sorts of epidemics have either been its precursors, have followed in its train, or have appeared in its outskirts! In England and France, for example, we have had the *grippe*, the epidemic cough of July last, and the affection of the bowels of August and September. Other and various epidemics are spoken of in other places in Europe and Asia.

"Let me now proceed to compare the atmospheric phenomena with these disorders. In January last, I believe on the night of the 7th, the most remarkable aurora borealis ever witnessed in England was seen the whole of the night; the whole of the north-east seemed of the colour of blood, while yellow, blue, and green, light, was conspicuous in the streamers to the south and east. A still more remarkable aurora was seen in Europe on the 11th of August, and strange colours, and unusual refractions in the sun's light, have been noticed all over Europe, while the whole of the present summer has been characterised by sudden changes from heat to cold, and vice versa, in various countries.

"A NEW VOLCANO *has broken out in the sea near Sicily!*

"On the 11th of August a tremendous tornado devastated the isle of Barbadoes, and other parts of the West Indies, and on the 11th of September an earthquake was felt at Venice and at Parma! These are, however, only a few of the remarkable phenomena that I could relate. Rivers have burst their bounds in various parts, and inundations have been severe on the continent; while daily accounts are arriving of similar facts in the Levant. I will not swell the catalogue." (P. 34.)

Merciful consideration, not to add to this catalogue of woes! we commend the determination; for, if such be causes, there are more than enough.

There is one paragraph, however, at nearly the commencement of the essay, which admits all that rational contagionists ever have contended for, viz. that cholera and other communicable diseases *may* be, not that they *must*, be, transmitted from one person to another. No contagionist ever doubts the primitive spontaneous origin of even the most contagious disorders: and few deny that such causes may again produce the like effects; all that is contended for with regard to cholera and other contagious diseases, is, that being present, whatever may have been their origin, they ARE *communicable*, and MAY *be communicated.*

*The Anatomy of the Horse, embracing the Structure of the Foot.* By WM. PERCIVALL, M.R.C.S., Veterinary Surgeon in the First Life Guards; author of "Lectures on the Veterinary Art," and co-editor of "The Veterinarian.—8vo. pp. 454. Longman and Co., London.

VETERINARY surgeons are now rapidly rescuing their profession from the hands of ignorant and unqualified pretenders: their art, which was once entirely in the hands of uneducated farriers and grooms, is now practised and taught as a science. Regular courses of lectures are given to students on the anatomy, physiology, and pathology of the horse, by veterinary teachers, whose general attainments and education are as honourable to themselves as they are creditable to their profession. We are exceedingly gratified at perceiving the zeal and talent of many veterinary surgeons of the present day, not merely on account of the advantages which hence arise to their own exclusive pursuit, but from a conviction that medical science in general will reap advantages from their labours and increased acquirements.

The work before us comes from the pen of a gentleman who is highly distinguished as a practitioner and a teacher. That such a treatise must have been much wanted, will be apparent when we state that, among the many veterinary works published in this country, this is the first exclusively dedicated to the anatomy of the horse; and we are assured by a very competent judge, to whom we have submitted it, that it is in every respect worthy the attention of the veterinary student. Mr. PERCIVALL has added to the utility of his work by consulting, on any doubtful points, the most eminent veterinary authorities, and particularly M. GIRARD, director of the Veterinary School at Paris. To Messrs. STUBBS, BEAN, and SMITH, he also acknowledges his obligations.

The veterinary student is now furnished with an anatomical guide upon which he may safely depend; as the structure of every part of the horse is clearly and correctly described by Mr. Percivall.

---

*The Nature and Treatment of the Epidemic Cholera.* By ROBERT VENABLES, A.M. &c. *Appendix: being a Numerical Abstract of Cases of Cholera in the different Stages of the Complaint, treated at Wick, &c.*—8vo. pp. 60.

THE opinions of Dr. VENABLES respecting the nature and treatment of epidemic cholera are already before the public, having appeared in his correspondence with the Board of Health; and we do not perceive, by these pamphlets, that he has found occasion to alter the opinions he had previously expressed. They are, therefore, not so valuable on account of any novelties they contain, as, by affording a clear and condensed statement of the conclusions at which an experienced, and apparently unbiassed, practitioner has arrived, after having ample opportunities for making observations.

This essay is divided into various sections and subsections, corresponding with the various stages of the disease, whether tending towards recovery or death: but through all these it is unnecessary that we should follow the author. Two or three short extracts, however, we shall make; and first with regard to the appearance of the eyes in the febrile stage.

"Whenever there is a transition to the febrile stage, there is always a dull languid expression of the eye, and it is invariably more or less suffused. The degree of suffusion is one of the most unerring means of prognosis with which I am acquainted. The moment febrile reaction takes place, I watch the appearance of the eye with the most scrupulous attention; for if suffusion to any extent or degree of severity set in, I look upon the case as lost. A fatal congestion in the brain is the result; and, although I have seen one or two recoveries in this hospital, under the circumstances above described, and as unpromising as it is well possible to imagine, yet I am not inclined at present to set a high value upon the remedial means adopted, notwithstanding the success which attended the activity and decision with which they are employed." (P. 9.)

Our second extract relates to the much canvassed and still unsettled point, viz. "the *causes of cholera.*"

"The predisposing are starvation, a poor watery diet, grief, anxiety, fear, drunkenness, and all sorts of intemperance, &c.; in short, any thing which tends to emaciate the body, enfeeble the frame, or depress the spirits. However, there is no one of these capable of itself of producing the disease, and therefore we must look for some more immediate exciting cause. There are but two which have been looked upon as capable of producing this disease, namely, malaria and contagion. My opinions upon these questions are already before the public, and I need, therefore, hardly again enter at length upon the subject: suffice it to say, that a very extensive and matured experience has only served to confirm the views which I have already advocated. The evidence which has traced the disease to contagion is as convincing to my mind, and proves the contagious character of the disease in the cold, the collapsed, and the typhoid, stages, as fully as such a subject will permit. However, I do not believe that either the preliminary diarrhœa or the premonitory stage are, generally speaking, contagious. In fact, contagion is not an integrant of febrile disease; but an accessory, the product of the morbid operations of the animal economy, while labouring for a time under the influence of fever. Thus, if a person be exposed to the contagion of typhus, he is seized with fever; but the commencement of that fever is not contagious, although it becomes contagious during the morbid operations of the animal economy. Therefore, contagion, according to this view, is not a primary, but an acquired, principle." (P. 20.)

The Appendix contains tabular views of the results of the author's practice, which, as they may form useful records, and be valuable for future reference, we shall transfer to our pages.

*Numerical Abstract of Cases of Cholera, in the different Stages treated at Wick, with the Results, &c.* (*Corrected to* 16*th October*, 1832.)

| Stages of the Disease at the period of visiting the patient.* | No. in each Stage. | DIED. In premonitory stage. | In cold stage. | In collapse. | In typhoid stage. | Total Deaths. | RECOVERED. By critical solution. | From cold stage. | From collapse. | From typhoid stage. | Total recoveries. | Remaining, but convalescent. | Average mortality of the different stages; the remaining patients being considered as entirely cured. |
|---|---|---|---|---|---|---|---|---|---|---|---|---|---|
| Premonitory stage, | 182 | 1 | 0 | 2 | 1 | 4 | 35 | 2 | 1 | 138 | 176 | 2 | $1.45\frac{1}{2}$ |
| Cold stage ......... | 70 | 0 | 3 | 4 | 3 | 10 | 11 | 0 | 2 | 45 | 58 | 2 | 1.7 |
| Collapse ............ | 52 | 0 | 0 | 24 | 18 | 42 | 0 | 0 | 3 | 7 | 10 | 0 | $4.4\frac{20}{21}$ |
| Typhoid stage...... | 13 | 0 | 0 | 0 | 3 | 3 | 0 | 0 | 0 | 9 | 9 | 1 | $1.4\frac{1}{3}$ |
| Totals ...... | 317 | 1 | 3 | 30 | 25 | 59 | 46 | 2 | 6 | 199 | 253 | 5 | $1.5\frac{22}{59}$ |

* These are independent of 879 bilious diarrhœas. From an examination of the druggist's accounts, I find the diarrhœas to have amounted to nearly 1800.

*Numerical Abstract of Cases of Cholera, in its different Stages, treated in the Cholera Hospital, Wick.* (*Corrected to* 16*th October*, 1832.)

| Stages at the period of admission into Cholera Hospital. | No. in each stage. | DIED. In premonitory stage. | In cold stage | In collapse. | In typhoid stage. | Total deaths. | RECOVERED. By critical solution. | From cold stage. | From collapse. | From typhoid stage. | Total recoveries. | Remaining under treatment this day. | Mortality in each stage, and average mortality of entire disease. |
|---|---|---|---|---|---|---|---|---|---|---|---|---|---|
| Premonitory stage, | 26 | 0 | 0 | 1 | 0 | 1 | 12 | 1 | 0 | 10 | 23 | 2 | 1.26 |
| Cold stage ......... | 29 | 0 | 0 | 3 | 1 | 4 | 6 | 2 | 0 | 15 | 23 | 2 | $1.7\frac{1}{4}$ |
| Collapse ............ | 36 | 0 | 0 | 18 | 5 | 23 | 0 | 0 | 0 | 13 | 13* | 0 | $1·1\frac{11}{23}$ |
| Typhoid stage...... | 4 | 0 | 0 | 0 | 0 | 0 | 0 | 0 | 0 | 3 | 3 | 1 | |
| Totals ......... | 95 | 0 | 0 | 22 | 6 | 28 | 18 | 3 | 0 | 41 | 62 | 5 | $1.3\frac{11}{28}$ |

* In hospital, the recoveries from collapse seem to exceed those in the general return; but this arises from the difficulty of distinguishing the termination of the cold from the beginning of the collapsed stage, as well as receiving them into hospital from the other practitioners.

